# Water Content Detection of Red Sandstone Based on Shock Acoustic Sensing and Convolutional Neural Network

**DOI:** 10.3390/s25237164

**Published:** 2025-11-24

**Authors:** Zhaokang Qiu, Yang Liu, Yi Zhang, Xueqi Zhao, Dongdong Chen, Shengwu Tu

**Affiliations:** 1 State Key Laboratory of Precision Blasting, Jianghan University, Wuhan 430056, China; 2 Hubei Key Laboratory of Blasting Engineering, Jianghan University, Wuhan 430056, China; 3 School of Resource and Environmental Engineering, Wuhan University of Science and Technology, Wuhan 430081, China; 4 School of Mechanical Engineering, Wuhan Polytechnic University, Wuhan 430023, China; 5 College of Civil Engineering, Nanjing Forestry University, Nanjing 210037, China

**Keywords:** red sandstone, knocking detection method, water content, Convolutional Neural Network

## Abstract

In response to the challenge of changes in the physical and mechanical properties of red sandstone when it comes into contact with water during construction projects, this paper proposes a moisture content detection method for red sandstone based on the knocking method. Taking red sandstone as the research object, this study explores a moisture content detection approach by combining the knocking method with Convolutional Neural Network and Support Vector Machine algorithms (CNN-SVM). Specifically, this research involves knocking the surface of red sandstone specimens with a knocking hammer and precisely capturing the acoustic signals generated during the knocking process using a microphone. Subsequently, an effective detection of the moisture content in red sandstone is achieved through a method based on feature extraction from knocking sound signals and a Convolutional Neural Network classification model. This method is easy to operate. By utilizing modern signal processing techniques combined with the CNN-SVM model, it enables accurate identification and non-destructive testing of the moisture content in red sandstone even with small sample datasets. Mel Frequency Cepstral Coefficients (MFCCs) and Continuous Wavelet Transform (CWT) were separately used as features for detecting red sandstone specimens with different moisture contents. The detection results show that the classification accuracy of red sandstone moisture content using MFCCs as the feature reaches as high as 94.4%, significantly outperforming the classification method using CWT as the feature. This study validates the effectiveness and reliability of the proposed method, providing a novel and efficient approach for rapid and non-destructive detection of the moisture content in red sandstone.

## 1. Introduction

As a typical sedimentary rock, red sandstone holds significant application value in fields such as transportation tunnel engineering, slope support, underground space development, and geotechnical engineering foundation treatment due to its widespread geographical distribution and unique physical and mechanical properties [1,2]. Studies have found that the engineering performance of red sandstone is highly dependent on its moisture content state, and the nonlinear impact of moisture content variation on the rock’s mechanical properties has emerged as a critical scientific issue constraining engineering safety [3,4]. Red sandstone with high moisture content is prone to causing tunnel face collapse and water and mud inrush accidents. Under the action of wetting–drying cycles, the moisture content of red sandstone fluctuates; a fluctuation range of 3% can lead to a 15–25% attenuation in shear strength, significantly increasing the risk of landslides [5]. The bearing capacity of red sandstone foundations with excessively high moisture content decreases substantially, necessitating improvement through drainage and reinforcement techniques [6]. Existing research has revealed the quantitative relationship between moisture content and rock properties through laboratory experiments and numerical simulations [7,8]. At low moisture content levels, the pore water film within rock is thin, and the cementation between mineral particles is strong, resulting in high hardness but significant brittleness. At moderate moisture content levels, water molecules exert a weakening effect between mineral layers, reducing hardness by 30–50%, but crack propagation paths are inhibited by water pressure, enhancing crack resistance by 20–30%. At high moisture content levels, clay minerals absorb water and expand, leading to the extension of micro-fractures; the rock’s volumetric expansion rate can reach 0.5–1.2%, while the internal friction angle decreases to below 25°, resulting in a loss of overall stability. In practical engineering projects, the moisture content of red sandstone affects its applicability, and it needs to be reasonably selected according to engineering requirements to ensure project quality and safety. Therefore, the detection of the moisture content of red sandstone is particularly crucial for ensuring its performance and stability.

At present, the detection methods for rock moisture content mainly include the dryer drying method, fiber optic sensor method [9], capacitance method [10,11], GPR method [12], acoustic emission method [13], etc. At present, these methods can achieve high accuracy in detecting rock moisture content, but they all have certain limitations. The dryer drying method consumes high energy, takes a long time to dry, and may cause certain damage to the rock. The fiber optic sensor method is limited by the fiber optic environment and has high requirements for the surface of the detected object, and the cost of this method is also high [14]. The temperature, density, and shape of rocks can affect the capacitance method based on dielectric constant measurement [15]. When electromagnetic waves encounter rocks, GPR devices receive signals reflected, refracted, or scattered back by the rocks. The water content of the rocks serves as a pivotal factor influencing the propagation speed of the received signals and is suitable for detecting the water content of red sandstone. However, this approach necessitates substantial time and financial investments [16]. The acoustic emission method requires the installation of sensors to obtain acoustic emission signals, which is costly and has limited coverage [17]. Hence, the quest for a simple, economical, efficient, and non-destructive approach to detect the water content within red sandstone.

The impact test has been studied for many years and is considered a non-destructive testing method [18]. Due to its simple operation, economy, and lightweight testing tools, impact detection for structural inspection has garnered widespread adoption in traditional impact diagnosis. However, inspection engineers focus more on the analysis of structural vibration response induced by knocking on the structure, while ignoring the sound caused by knocking. In recent years, the sound caused by knocking has received increasing attention from researchers. For example, Yuan et al. [19] detected the moisture content of wood by knocking its surface and compared the detection results of the random forest classifier to classify the features extracted from the wavelet packet decomposition method (RF+WPD) and Mel Frequency Cepstral Coefficients combination with Convolutional Neural Network (MFCCs+CNN) algorithms. He et al. [20] manually tapped the pipeline from different sides and recorded the sound signals of the pipeline under different sand-to-water ratios using a microphone. They classified the large amount of sandy sediment in the pipeline using an SVM algorithm. Chen et al. [21] used a hammer to strike each non-void and void sub-region in the filled steel pipe, processed the recorded sound signal density using power spectrum processing, and compared the prediction accuracy using Support Vector Machine (SVM) and decision tree classification methods. Yang et al. [22] remotely operated the vehicle connection hardware for pinpointing damage to submerged concrete structures. Using the knocking method, the hydrophone gauges the knocking response and regulates the system’s influence on the structural surface. After denoising and signal separation, damage indicators are extracted from the processed signal to accurately identify the damage status. Zheng et al. [23] introduced a knocking-based technique for concrete moisture detection. The concrete sample’s surface was impacted with a hammer, and the resultant knocking sound was captured by a microphone for analysis. After processing and analyzing audio data, using Support Vector Machines to accurately identify and classify the processed data can assess the water content within concrete.

The most commonly used speech feature in speech recognition and speaker recognition is the MFCC, which has been extensively employed in the field of voice recognition [24]. Some researchers have developed a knocking-based method to classify wood using the MFCC as a feature of knocking sound using CNN, in order to identify the moisture content of wood. The findings suggest that the knocking-based method exhibits high precision in determining the water content within wood [17]. However, there is little research on using knocking to detect the water content within red sandstone.

This paper introduces a novel methodology for detecting the water content within red sandstone, which is based on the principle of the knocking method. By precisely controlling the immersion duration of red sandstone specimens in water, specimens with varying degrees of water content can be obtained. Subsequently, a knocking hammer was used to strike the surface of the red sandstone sample, and a microphone was used to accurately capture the sound signal generated by the strike. In order to analyze these sound signals more deeply, this article adopts MFCC technology to convert the knocking induction signals at different water contents into frequency domain features. Furthermore, key features are extracted from the MFCC parameter map and fed into the CNN framework. In the model training phase, we utilized the Adaptive Moment Estimation (Adam) optimization technique to enhance both the training efficiency and the predictive accuracy of our model. After training, the SVM algorithm was used to classify the model, thereby achieving accurate recognition of the water content within red sandstone. Compared with other approaches for detecting the water content in red sandstone samples, this knocking detection method is not only easy to operate, but also has the advantage of non-destructive testing, so it has broad application prospects in related fields.

This article is organized in the subsequent sections as outlined below: Section 2 elaborates on the basic principle and calculation steps of MFCC, analyzes the operation mechanism and workflow of CNN, clarifies the definition of SVM, and explores the role and selection of its kernel function. On this basis, a thorough study was conducted on the definition of the Convolutional Neural Network combination with Support Vector Machine (CNN-SVM) model, and the techniques and benefits of integrating these two methods were elucidated. Section 3 focuses on the preparation and data collection process of red sandstone samples, introducing the selection criteria, processing steps, and data collection process of the samples. Section 4 comprehensively analyzes the experimental data, summarizes the trend of water content of red sandstone samples changing with environmental conditions, and evaluates the precision performance of this method in water content detection through quantitative analysis, highlighting the progressiveness and accuracy of the technology. In Section 5, the experimental results obtained by knocking to detect the water content of red sandstone are presented, and the possible limitations of this method in practical applications are analyzed. Meanwhile, looking forward to future research directions and proposing improvement plans. Section 6 serves as a summary of the entire article, reiterating the main findings and conclusions of the experiment. In addition, the potential practical significance of this method in the field of red sandstone water content detection was emphasized.

## 2. Materials and Methods

### 2.1. Detection Principle

**Figure 1:**
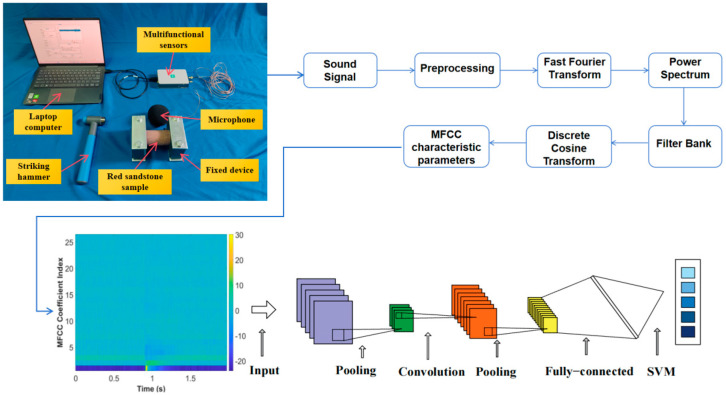
Data extraction and processing.

The process of proposing the method is shown in Figure 1.

(1) Obtain a knocking sound signal: Use a knocking hammer to tap the side of the red sandstone sample, record the sound generated by the knocking through a microphone, and save the obtained sound signal in a laptop computer.
(2) Extracting frequency domain features of knocking sound signals through MFCCs can be mainly divided into five steps: pre-processing, fast Fourier transform, power spectrum, filter bank, discrete cosine transform, etc.
(3) Input the obtained MFCC features into CNN training: CNN extracts abstract features from the raw data through multi-layer convolution and pooling operations, and then uses the Adaptive Moment Estimation optimization algorithm to train the proposed model.
(4) Using SVM for image classification: The SVM model incorporates the fully connected layer of CNN as its input, enhancing the training of feature vectors, classification, and decision-making processes.

### 2.2. MFCC

In the fields of speech recognition and speaker recognition, the MFCC is one of the most commonly used and core speech features [25,26]. According to research findings on the auditory mechanism of the human ear, it has been discovered that the human ear exhibits varying degrees of auditory sensitivity to sound waves of different frequencies; in the low-frequency region below 1000 Hz, there is a roughly linear relationship between sensitivity and frequency. Once the frequency exceeds 1000 Hz and enters the high-frequency region, this relationship changes into a logarithmic relationship, meaning that as the frequency increases, the growth rate of human ear sensitivity gradually slows down [27]. The Mel Cepstral Coefficient, a pivotal acoustic feature using the Mel frequency scale, effectively mimics the nonlinear frequency perception of the human ear [28]. The correspondence between Mel frequency and physical frequency can be mathematically expressed using the following approximation formula:

(1) $$\mathrm{Mel}(f)\;=\;2595\mathrm{lg}(1+\frac{f}{700}),$$

In the formula, Mel(f) represents the Mel scale frequency, with f denoting the frequency in Hz.

The extraction of speech feature parameters MFCCs mainly includes five processes [29]: pre-processing, which includes pre-emphasis, framing, and windowing; application of fast Fourier transform (FFT); power spectrum; utilization of filter bank; and, finally, execution of discrete cosine transform (DCT).

Due to the fact that low-frequency sound travels a longer distance on the inner cochlear basement membrane compared to high-frequency sound, it is generally easier for low frequencies to mask high frequencies, while high frequencies are more difficult to mask low frequencies. Pre-emphasis aims to enhance the high-frequency components of the audio, flatten the frequency spectrum of the sound signal, and maintain the frequency band throughout the entire process from low to high frequencies. Adopting an identical signal-to-noise ratio metric allows us to compute the frequency spectrum [30]. Additionally, pre-emphasis processing essentially involves filtering the speech signal via a high-pass filter:

H(z) = 1 − μz^−1^ (2)

In the formula, H(z) is the filter function, where z signifies the signal’s frequency, and μ denotes the value of the filter coefficients, which are usually 0.95 or 0.97 [31].

**Figure 2:**
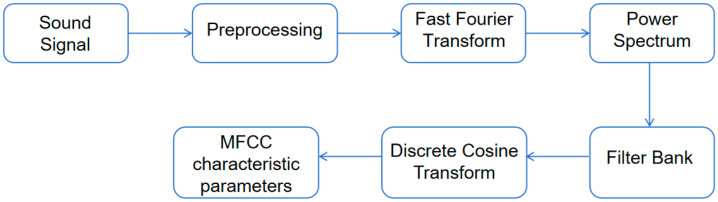
MFCC workflow.

After pre-emphasis processing, in order to cope with the non-stationarity of the signal and utilize its short-term stationarity, frame division technology is usually used to cut the signal into a series of continuous short-term frames. Meanwhile, to minimize notable variations across consecutive frames, an overlapping region is established between adjacent frames. After segmenting the signal into frames, in order to alleviate spectrum leakage, each frame undergoes the application of a Hamming window function, enhancing the continuity between its left and right extremities [32]. Due to the intricacies of identifying signal features in the time domain, the FFT transformation is commonly performed to visualize the distribution of energy across the frequency spectrum [33]. Following the FFT transformation, the squared frequency spectrum of the speech signal is calculated to derive its spectral line energy. To diminish the disruption from harmonic components, each frame’s spectrum is smoothed utilizing a set of triangular filters adhering to the Mel scale, and highlighting the resonance peaks of the original speech. Ultimately, the filter bank’s energy undergoes a logarithmic transformation, and a discrete cosine transform is applied to decorrelate the filter bank coefficients, resulting in the extraction of MFCCs [29]. This entire computation procedure is visually depicted in Figure 2.

### 2.3. CNN-SVM

#### 2.3.1. CNN

**Figure 3:**
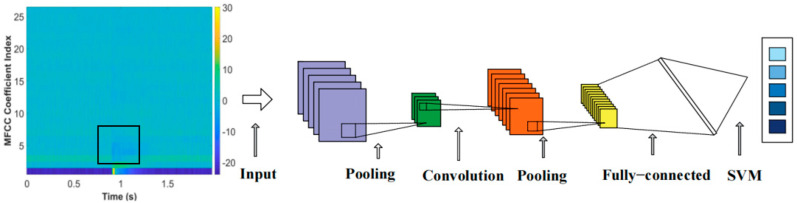
Convolutional Neural Network workflow.

CNN, a deep learning architecture akin to multi-layer perceptrons in artificial neural networks, excels in analyzing visual images and has garnered widespread adoption in domains encompassing computer vision and language recognition [34,35]. The CNN’s feature extraction layer implicitly acquires knowledge from training data, bypassing the need for explicit feature extraction, and, instead, harnessing MFCC training data to discern latent features, which is a method to enhance prediction accuracy [36]. As shown in Figure 3, the standard architecture of a Convolutional Neural Network typically encompasses five fundamental components: an input layer, convolutional layers, pooling layers, fully connected layers, and an output layer. Firstly, the input layer is responsible for receiving information, such as images, as the starting point of the network. Subsequently, the convolutional layer scans the input image using convolutional kernels to identify and locate specific feature regions in the image, and generates feature maps based on this to predict the classification to which these features belong. Following the convolution process, the pooling layer performs further feature extraction and compression on the obtained feature maps. It selects and retains the most representative features, thereby addressing the issue of an excessive number of features that may arise from the convolutional layer. The fully connected layer consolidates the features from the convolutional and pooling layers, performing flattening and summation operations on these features, and ultimately mapping these features to the corresponding classification labels, generating a probability value to indicate the likelihood that the image belongs to various categories [37,38].

In the multi-classification task of deep learning, the output layer plays a crucial role. It receives the original output from the fully connected layer, performs final transformation and processing on these outputs, calculates the probability value of each classification category, and outputs the final classification result [39].

#### 2.3.2. SVM

The SVM algorithm originates from statistical learning theory, and its core lies in the principle of minimizing structural risk. Within the realm of machine learning, SVM has attracted much attention for its excellent performance and wide applicability when dealing with classification problems [40,41].

The core concept of SVM revolves around identifying an optimal hyperplane that functions as a decisive barrier, effectively segregating the training data into distinct groups, thereby guaranteeing that data points belonging to different classes are positioned on opposite sides of this hyperplane. Meanwhile, by maximizing the spacing between data points situated on either side of the hyperplane and maintaining the minimum distance from the hyperplane (these points are called support vectors), a robust and accurate classifier is constructed [42].

For a set of training sets Q = {($x_{1}$,$y_{1}$), ($x_{2}$,$y_{2}$),…, ($x_{N}$,$y_{N}$)}, among them, xi∈Rn denotes the characteristic vector pertaining to the i-th instance, whereas $y_{i}$∈{+1,−1} signifies the label of the data point, used to indicate its category. For i = 1, 2,…, N. Assuming the data is linearly separable, there exists a hyperplane H:

H = ωx + b = 0,(3)

In the formula, ω is the hyperplane normal vector, which determines the direction of the hyperplane; x represents a coordinate on the hyperplane; and b denotes the intercept.

The set interval from each sample point ($x_{i}$,$y_{i}$) in the training set to the hyperplane is

(4) $$y_{i}=\;y_{i}\left(\frac{\omega }{{\left|\left|\omega \right|\right|}_{y}}\times x_{i}+\frac{b}{\left|\left|\omega \right|\right|}\right)$$

To solve the hyperplane problem that maximizes the segmentation distance (i.e., interval), can achieve the goal by solving the following optimization problem in the form of the following:

(5) $$\max\;y\;s.t.y_{i}=\;y_{i}\left(\frac{\omega }{{\left|\left|\omega \right|\right|}_{y}}\times x_{i}+\frac{b}{{\left|\left|\omega \right|\right|}_{y}}\right)\geq 1(i=1,2,3,\cdots ,N),$$

Let $\omega =\frac{\omega }{{\left|\left|\omega \right|\right|}_{y}}$, $b=\frac{b}{{\left|\left|\omega \right|\right|}_{y}}$, and, because maximizing γ can be transformed into minimizing $\frac{1}{2}{\left|\left|\omega \right|\right|}^{2}$ through further mathematical transformation, the final classification decision function [43] can be obtained as follows:

(6) $$f(x)=\;sgn\{\sum_{i=1}a_{i}^{*}y_{i}(x_{i}fx_{j})+b^{*}\},$$

In the formula: $a_{i}^{*}$ for support vectors, $x_{i}fx_{j}$ for kernel functions. In the architecture design of SVM, a series of diverse kernel functions are adopted, aimed at projecting the raw data into a high-dimensional feature space, thereby bolstering the model’s classification proficiency. The techniques used in this process include, but are not limited to, polynomial kernel functions, linear kernel functions, Gaussian radial basis functions (RBF), and sigmoid kernel functions. Each kernel function has its unique properties and applicable scenarios, enabling flexible customization tailored to the dataset’s distinct distributions and classification needs [44], as detailed in Table 1. Notably, parameters G for the kernel function and C for the penalty play a crucial role in optimizing SVM classification performance, and their effective adjustment can significantly improve classification accuracy [45].

#### 2.3.3. Fusion of CNN and SVM

The traditional CNN classification layer usually relies on the design of a combination of fully connected layers and Softmax classifiers. However, there are two main challenges to this structure. Firstly, the fully connected layer is prone to overfitting due to its large amount of data, which implies that the model exhibits an overly precise performance on the training data, to the extent that it learns noise or specificity from the training data, resulting in a decrease in generalization ability on unseen test data, and performance may not be as expected. Secondly, although Softmax classifiers are a commonly used choice when dealing with multi-classification problems, their performance may not be as good as SVM in some complex multi-classification scenarios, exhibiting enhanced robustness, adeptly handling high-dimensional data alongside the intricacies inherent in complex classification boundaries [46].

The CNN-SVM method ingeniously combines the powerful feature extraction capability of CNN and the excellent classification performance of SVM. CNN extracts abstract features from raw data through multi-layer convolutional and pooling operations. The convolutional layers utilize convolutional kernels to scan the input signals and generate feature maps. The pooling layers further compress the features while retaining the most representative information, and these features are then fed into the SVM for classification. As a classifier, SVM receives the feature vectors extracted by CNN and searches for the optimal hyperplane based on the principle of maximizing the margin, thereby achieving accurate classification of moisture content. The key to this method lies in the fact that CNN can effectively learn useful features from the data, while SVM can accurately classify based on these features. This classification approach enhances the model’s flexibility and accuracy [47].

### 2.4. CWT (Continuous Wavelet Transform)

The basic principle of wavelet time–frequency analysis (or wavelet transform time–frequency graph) is to use wavelet transform as a mathematical tool to finely decompose and display signals in both time and frequency dimensions, so as to clearly reveal the specific characteristics of signals at different times and frequencies [48]. The key lies in using the scaling and translation of wavelet functions to match different frequency components in the signal, and calculating corresponding wavelet coefficients, mainly involving CWT and scale to frequency conversion [49], which can be formulated as

(7) $$\left\{f(t),\psi_{p,q}(t)\right\}=\frac{1}{\sqrt{p}}\int_{-\infty }^{+\infty }x(t)\psi (\frac{t-q}{p})\mathrm{dt},$$

In the formula, x(t) denotes the signal to be analyzed, ψ(t) represents the basic wavelet function, p denotes the factor for scaling, and q signifies the factor for translation.

## 3. Experimental Setup and Procedures

### 3.1. Water Content of Red Sandstone Samples

**Figure 4:**
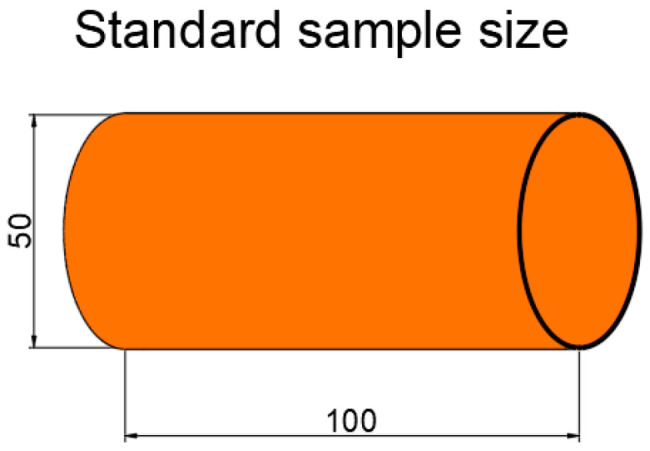
Sample preparation.

**Figure 5:**
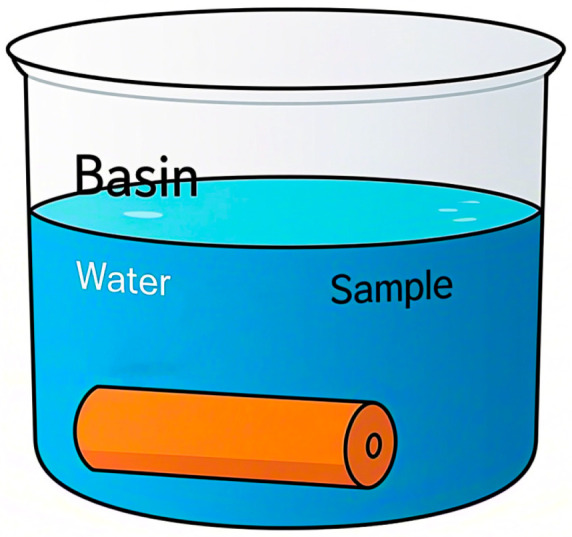
Sample soaking.

To verify the effectiveness of the knocking method based on MFCCs in detecting the moisture content of red sandstone, as shown in Figure 4, we prepared a cylindrical red sandstone specimen (Zhongmin Stone, Wuhan, China) with dimensions of 50 × 100 mm. Some of the physical and mechanical properties of the specimen are presented in Table 2. Initially, the sample was placed in an oven at 105 °C for continuous drying over 8 h until its dry weight reached a constant state, ensuring that the measurement error was controlled within a precise range of 0.5% during this process. Subsequently, to simulate different moisture content conditions, these dried specimens were individually soaked in water, as depicted in Figure 5, with the soaking times conducted according to the data listed in Table 3.

**Figure 6:**
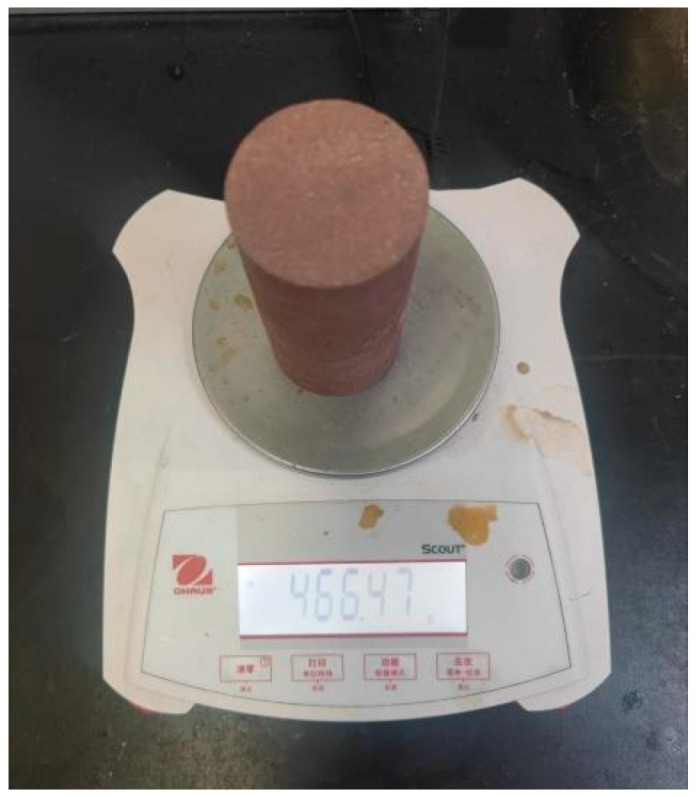
Sample weighing.

After the soaking is completed, remove the specimen from the water and carefully wipe it with a dry cloth to ensure that there are no residual water droplets on the specimen’s surface. Subsequently, weigh the treated specimen using an electronic scale (Fujian Qiaoan Electronic Technology Co., Ltd., Quanzhou, China), as shown in Figure 6. To obtain more accurate data, the aforementioned processes of soaking, wiping, and weighing were repeated, with a total of 12 cycles of operations conducted on each specimen. Through these detailed steps, key data for validating the effectiveness of the MFCC-based knocking method were collected.

After each weighing, a hammer is used to strike the specimen to elicit an acoustic signal, which is then collected by a microphone. Subsequently, the specimen is returned to the water for soaking. During the striking process, owing to the short duration, the moisture content within the red sandstone is considered to be constant. The moisture content of the red sandstone is calculated according to the following formula:

(8) $$\omega^{\prime }=\frac{m-m_{dr}}{m_{dr}}\times 100\%,$$

In the formula, ω^′^ is the water content within red sandstone, m represents the total mass including water, and $m_{dr}$ denotes the mass after drying.

### 3.2. Data Collection Process

**Figure 7:**
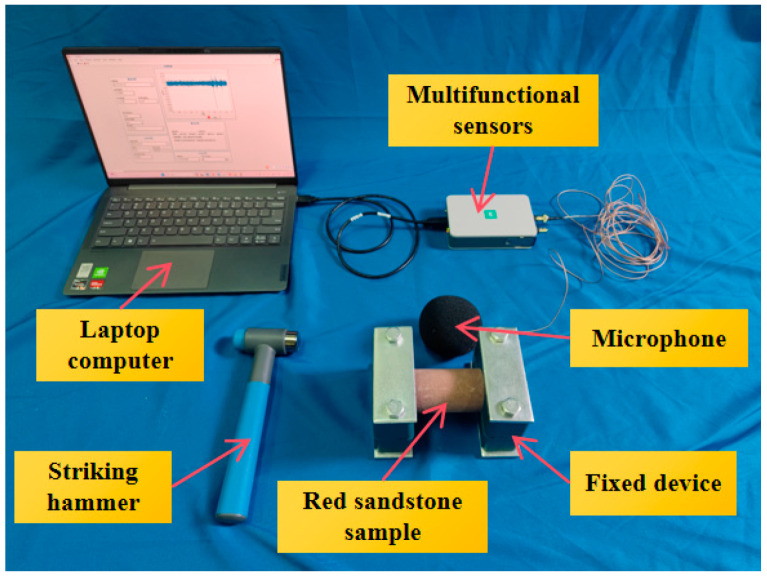
Schematic diagram of experimental setup.

The experimental setup is shown in Figure 7. In the experiment, both ends of the specimen were fixed using a fixing device. Then, a hammer was used to strike the side surface of the red sandstone specimen. The sound signals generated from the striking were recorded via a microphone, transmitted through a multifunctional I/O device (National Instruments, Austin, TX, USA), and saved on a laptop running on the LabVIEW (Version 2018)operating platform. Based on the changing trend of the specimen’s moisture content over time, and to better differentiate the moisture content levels of the specimens, this experiment conducted 100 strikes on each group of specimens under five different moisture content conditions (dry, soaked for 10 min, soaked for 20 min, soaked for 40 min, and soaked for 240 min) for the red sandstone specimens. The sampling frequency was set at 51.2 kHz.

## 4. Result

**Figure 8:**
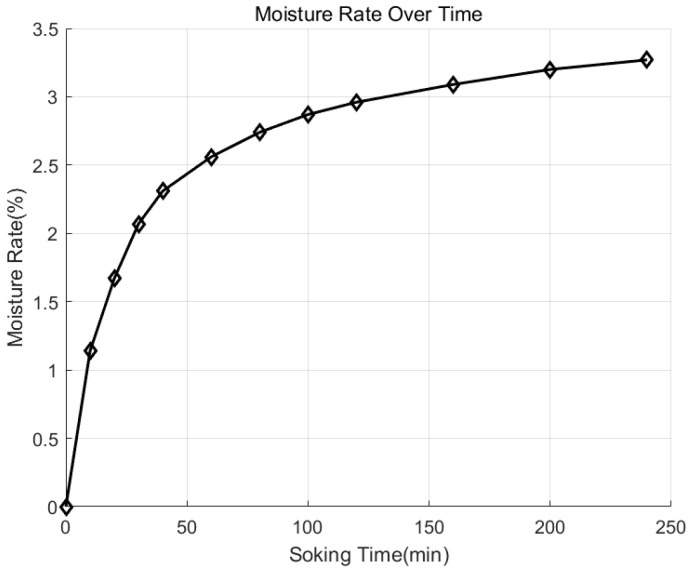
The relationship between immersion time and sample moisture content.

**Figure 9:**
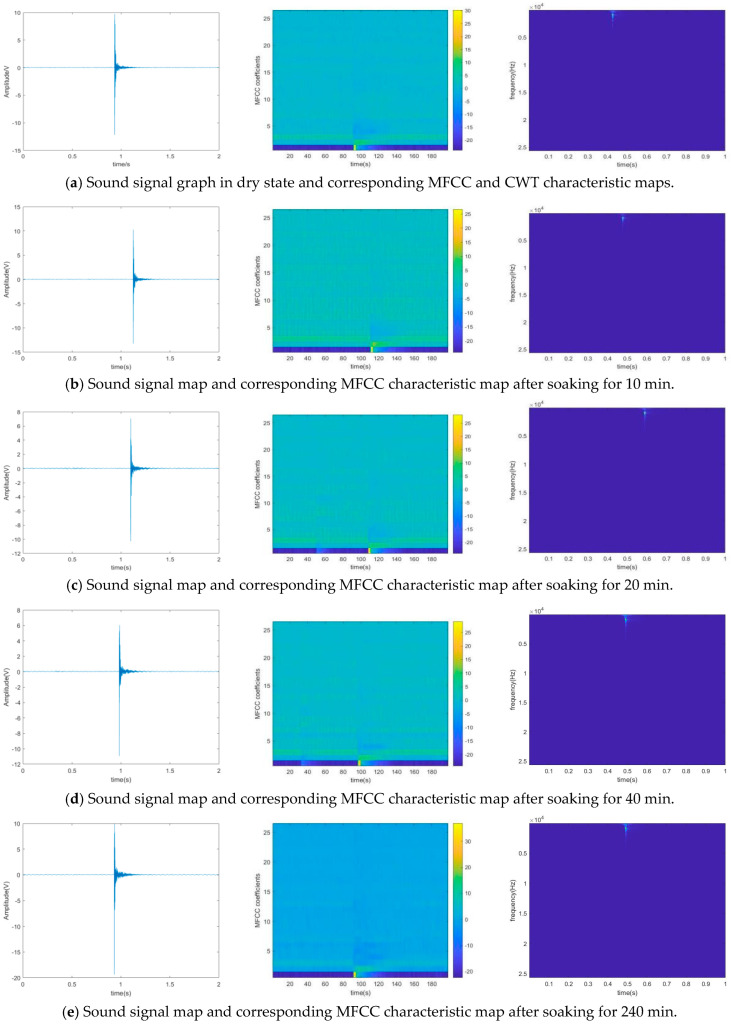
Knocking sound signal and corresponding MFCC and CWT feature maps.

As shown in Figure 8, the relationship between the red sandstone sample and its soaking time in water can be clearly seen from the figure, and the water content of the red sandstone sample shows a significant increasing trend. The trend changes quickly within 50 min, and the growth trend of water content decelerates steadily after 50 min. The knocking signal recorded by the microphone and the processed MFCC and CWT feature maps are shown in Figure 9.

**Figure 10:**
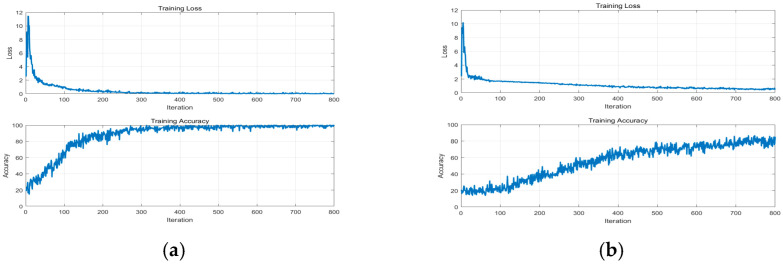
Accuracy and loss rate chart of MFCC: (**a**) MFCC, (**b**) CWT.

**Figure 11:**
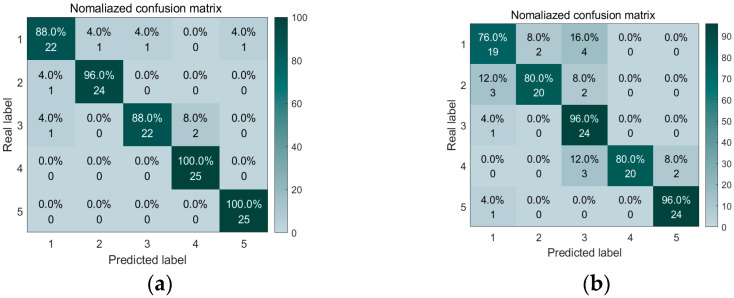
Confusion matrix diagram of two acoustic features: (**a**) MFCC, (**b**) CWT.

**Figure 12:**
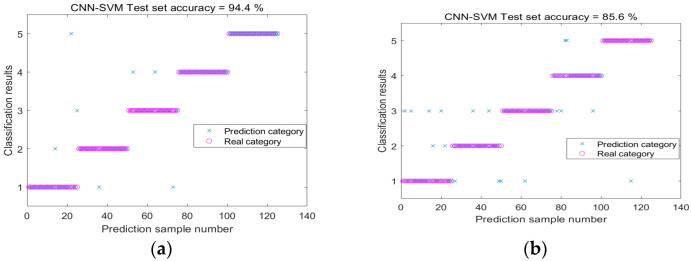
Accuracy maps of CNN-SVM validation sets for two types of feature maps: (**a**) MFCC, (**b**) CWT.

In order to accurately extract the information of these feature maps under different water content conditions, we constructed a Convolutional Neural Network recognition model containing four continuous convolutional blocks. During the training process, we adopted the Adaptive Moment Estimation optimization algorithm and configured the learning rate to 0.001 with an iteration period of 200 times. Figure 10 illustrates the changes in training and validation accuracy as well as loss rate across 500 samples in the entire dataset. Notably, the curves for MFCCs exhibit greater smoothness and faster convergence, indicating their high efficiency in feature extraction and classification. This further demonstrates the superiority of MFCCs in processing knocking sound signals of red sandstone. Upon analyzing the confusion matrix in Figure 11a, one can observe that when MFCCs are used as features, the classification accuracy of the samples soaked for 40 min and 240 min is 100%, the accuracy of the samples soaked for 10 min is 96%, and 4% is the corresponding error rate. Figure 12 compares the training accuracy using two acoustic features, MFCCs and CWT. Among them, the validation accuracy based on MFCCs is 94.4%, significantly higher than the 85.6% based on CWT; this result can be attributed to the fact that MFCCs can better simulate the nonlinear perceptual characteristics of the human ear towards sound frequencies, thereby capturing the subtle sound differences caused by changes in the moisture content of red sandstone. In contrast, although CWT can provide detailed information about a signal in both time and frequency domains, it may not effectively distinguish the subtle variations caused by moisture content when processing complex sound signals. Table 4 presents the evaluation metrics of prediction results under different measured moisture content values, including precision, recall, F1-score, and their corresponding states. In the dry state, all metrics perform well. As the moisture content changes to states such as short-term water absorption and mid-term water absorption, although there are fluctuations in metrics like precision, they generally remain at a high level. These results validate the superiority of the CNN-SVM model proposed in this paper in the red sandstone moisture content classification task with MFCCs as the feature.

## 5. Discussion

In this study, we propose a water content detection scheme for red sandstone that does not require special equipment and is easy to operate. This scheme uses a hammer to strike red sandstone samples of varying water contents, and uses a microphone to capture and record the sound produced by the knocking. Importantly, although the knocking force varies, our primary focus lies on the variation in sound signal amplitude, as observed in the frequency domain, for each knocking event. After in-depth analysis and experimental results verification, the findings confirm that varying impact forces do not compromise the classification accuracy.

The following are several key findings of this study:

(1) Acoustic feature comparison: We compared the training accuracy using two acoustic features, MFCCs and CWT. Utilizing MFCCs as features, our experiments achieved a validation accuracy of 94.4%, significantly higher than the 85.6% using CWT as features, this result can be attributed to the fact that MFCCs can better simulate the nonlinear perceptual characteristics of the human ear towards sound frequencies, thereby capturing the subtle sound differences caused by changes in the moisture content of red sandstone. In contrast, although CWT can provide detailed information about signals in both time and frequency domains, it may not effectively distinguish the subtle variations caused by moisture content when processing complex sound signals, indicating that MFCCs have higher accuracy in processing such sound signals.
(2) Advantages of the proposed method: As a non-destructive testing technique, the knocking method, when combined with the MFCCs and CNN-SVM model, enables moisture content detection without damaging rock samples, achieving high-precision classification of moisture content and providing reliable technical support for engineering practice. Compared to traditional detection methods, the knocking method is easy to operate, requires no complex equipment, and is suitable for rapid on-site testing, which is particularly important for precious or non-renewable rock samples.
(3) Limitations of practical application: Although our detection scheme has significant advantages, there are also some limitations in practical applications. Firstly, the shape, size, and geological characteristics of the sample may cause changes in the knocking sound, which in turn affects the reliability of the detection method. Secondly, this study was conducted in a relatively quiet environment, whereas noise present in real-world settings may interfere with the acquisition and processing of knocking sound signals, thereby affecting classification accuracy. Despite its excellent performance in laboratory conditions, the model’s generalization capability across different environments and rock types still requires further validation.
(4) Future research direction: To enhance the accuracy and applicability of the detection scheme, we plan to make the following improvements in our future work. Firstly, we will increase the categories and quantity of samples to cover a broader range of red sandstone types and moisture content levels. Secondly, we will develop a noise reduction algorithm tailored for knocking sound signals and conduct experiments under various noise environments to evaluate the impact of different noise levels on detection results, thereby improving detection accuracy in noisy settings. Finally, we will further optimize the architecture of the CNN-SVM model to enhance the efficiency of feature extraction and classification and strengthen the model’s generalization capability. Through these measures, we aim to further refine and optimize the red sandstone moisture content detection scheme based on the knocking method. Additionally, we will extend this approach to other types of rocks or materials, such as sandstone, shale, concrete, etc., to assess its applicability across different materials.

## 6. Conclusions

Red sandstone, a common rock type, is found worldwide, and its water content is directly related to its mechanical properties and stability. In order to accurately determine the water content of red sandstone, this study innovatively proposes a non-destructive testing technology scheme based on the impact principle. Used a microphone to capture the sound signal generated by knocking, and compared the performance of MFCCs and CWT in feature extraction of sound signals. We found that MFCCs performed better in identifying samples of red sandstone with different water contents. These extracted features are then input into the CNN architecture, and the model is finely trained using the Adam optimization algorithm. After training, SVM was used as a classifier to accurately identify the water content of red sandstone.

The experimental data shows that the accuracy of water content classification for red sandstone characterized by MFCCs is as high as 94.4%, significantly better than CWT. The knocking method, as an intuitive and simple detection method, has shown its potential application value in the field of red sandstone water content detection. However, we also realize that there is still room for improvement in the accuracy and generalization ability of this method.

In future research, we will delve deeper into the influence of the structural size, shape, and environmental factors of different rock samples on detection methods and optimize our model accordingly. In addition, considering the manual knocking method currently used in research, we will explore the possibility of automated intelligent knocking systems to achieve more efficient and accurate detection of rock moisture content.

## Figures and Tables

**Table 1:** Some commonly used kernel functions.

Kernel Function	Equation
Polynomial function	$x_{i}fx_{j}={\left(x_{i}x_{j}+C\right)}^{d}$
Linear function	$x_{i}fx_{j}=x_{i}x_{j}$
Gaussian radial basis function	$x_{i}fx_{j}=\exp\left(G||x_{i}{-x}_{j}{\left|\right|}^{2}\right)$
Sigmoid ernel functions	$x_{i}fx_{j}=\tanh\left(G||x_{i}{-x}_{j}{\left|\right|}^{2}\right)$

**Table 2:** Mechanical and physical properties of test samples.

Characteristic	Numeric Cange
Unit weight (kN/m^3^)	22–25
Uniaxial compressive strength (MPa)	20–60
Porosity (%)	10–25
Elastic modulus (GPa)	5–20
Poisson’s ratio (ν)	0.2–0.35

**Table 3:** Immersion time of samples.

Sample	Immersion Time/min											
1	0	10	20	30	40	60	80	100	120	160	200	240

**Table 4:** Comparison of Prediction Results.

Measurement of Moisture Content(%)	Precision(%)	Recall(%)	F1-Score(%)	State
0.00	100.00	92.00	95.83	Dry state
1.14	100.00	96.00	97.96	Short-term water absorption
1.67	92.00	92.00	92.00	Mid-term water absorption
2.31	92.59	100.00	96.15	Approaching saturation
3.27	96.15	100.00	98.04	Approaching fully saturated

## Data Availability

The data presented in this study are available upon request from the corresponding author.
